# Adolescent Pregnancy: A Comparative Insight into the Prevalence and Risks of Obstetric Complications in a Polish Cohort

**DOI:** 10.3390/jcm13195785

**Published:** 2024-09-28

**Authors:** Jakub Staniczek, Maisa Manasar-Dyrbuś, Rafał Stojko, Cecylia Jendyk, Marcin Sadłocha, Ewa Winkowska, Dominika Orszulak, Kacper Niziński, Kaja Skowronek, Jakub Toczek, Aleksandra Matonóg, Katarzyna Wilk, Maja Zięba-Domalik, Diana Sieroszewska, Aleksander Sieroszewski, Joanna Starczewska, Daria Sowa-Sanchez, Jakub Jurecki, Jonasz Troszka, Szymon Stojko, Agnieszka Drosdzol-Cop

**Affiliations:** 1 Chair and Clinical Department of Gynecology, Obstetrics and Gynecological Oncology, The Medical University of Silesia in Katowice, Markiefki 87, 40-211 Katowice, Poland; maisamanasar@gmail.com (M.M.-D.);; 2 Department of Gynecology, Obstetrics, Gynecological Oncology, Pediatric and Adolescent Gynecology, Bonifraters’ Medical Center, Markiefki 87, 40-211 Katowice, Poland; 3 Institute of Health Sciences, University of Opole, 45-060 Opole, Poland

**Keywords:** adolescent pregnancy, teenage pregnancy, obstetrics complications, birth

## Abstract

**Background:** Adolescent pregnancy is associated with increased risk of both maternal and neonatal complications. Common maternal complications include anemia, hypertensive disorders, and a higher incidence of infections, including Group B Streptococcus (GBS). Additionally, adolescents are at increased risk for gestational diabetes and postpartum hemorrhage. Neonatal complications often involve low birth weight, prematurity, and an increased likelihood of neonatal intensive care unit (NICU) admission. **Objectives:** This study aims to assess and compare the prevalence of obstetric complications between adolescent and older pregnant women. **Methods:** This retrospective study investigates obstetric outcomes in adolescent pregnancies, analyzing data collected from 1 January 2016 to 30 June 2024. This study included 902 participants, of whom 224 were adolescents. The variables were categorized into maternal, birth, and neonatal characteristics. **Results:** Adolescent patients demonstrated a significantly higher prevalence of Group B Streptococcus (GBS) infection, affecting 25.89% of this group. Adolescent patients reported 17.86% nicotine use during pregnancy, a rate significantly higher than that of older age groups (*p* < 0.001). Additionally, adolescent pregnancies were associated with the highest mean blood loss during delivery, averaging 500 mL during vaginal birth and 1050 mL during cesarean section, leading to a higher incidence of blood transfusions (3.13%, *p* = 0.021). Newborns from adolescent pregnancies had the lowest mean birth weight (3199 g) and length (53.6 cm). Neonatal complications were more frequent in this group, affecting 20.09% of newborns, with a significantly higher rate of admission to intensive care units (2.68%, *p* = 0.008). These findings underscore the need for targeted interventions and more proactive management strategies to address the specific challenges faced by this population.

## 1. Introduction

### 1.1. Backround

Adolescent pregnancy is associated with significant risks for both maternal and neonatal health. Studies have shown that neonates born to mothers aged 10–19 years are at higher risk of severe complications, including low birth weight, prematurity, and low Apgar scores [1,2,3]. It was also found that adolescent pregnancies more frequently required neonatal intensive care compared to those born to older mothers [4,5]. These complications contribute to a higher burden of neonatal morbidity and mortality. For instance, neonatal complications were present in 68% of cases involving adolescent mothers [6].

Low birth weight, one of the most common issues among neonates of adolescent mothers, is often a result of fetal growth restriction (FGR). The study by Kakoo Brioso et al. highlighted that FGR is particularly prevalent in this group, potentially leading to long-term health issues, including physical and cognitive developmental delays [7]. Additionally, oligohydramnios is more commonly diagnosed in adolescent pregnancies, increasing the risk of perinatal complications such as meconium aspiration syndrome or fetal distress [4,7].

Another significant concern is the increased risk of preterm birth. Azevedo et al. found that adolescent mothers have a higher likelihood of delivering before 37 weeks of gestation, which substantially raises the risk of neonatal complications, such as respiratory distress syndrome, intraventricular hemorrhage, and retinopathy of prematurity [8]. Prematurity is also associated with prolonged stays in the neonatal intensive care unit (NICU), which further escalates healthcare costs and imposes an emotional strain on the young mother. Another reason for frequent NICU admissions in pregnancies among adolescents is maternal anemia, which occurs with prevalence rates ranging from 13.9% to 41.27% [8,9].

It is also noteworthy that despite some favorable outcomes, such a reduced need for cesarean section, likely due to a lower prevalence of obesity in this population, neonatal complications remain a significant challenge [10,11]. Although cesarean deliveries are less frequent, there is still an increased risk of birth injuries, such as brachial plexus injury, necessitating careful management during delivery [9].

In conclusion, neonatal complications in adolescent pregnancies pose a substantial challenge to the healthcare system. They require specialized prenatal care and a tailored approach to pregnancy and delivery management. Medical interventions are crucial in mitigating risks and improving health outcomes for both the mother and the neonate [12]. Additionally, continuous research across diverse populations is necessary to analyze the frequency and risks of complications [13].

This ongoing research is the first in the Polish cohort and includes data from the only Adolescent and Pediatric Gynecology Department in Poland. The results of this study are essential for conducting precise and targeted monitoring of adolescent pregnancies, focusing on specific risks to improve both maternal and neonatal outcomes.

### 1.2. Objectives

The main objective of this study is to assess and compare the prevalence of obstetric complications between adolescent and older pregnant women.

## 2. Methodology

### 2.1. Study Design

This retrospective study aims to investigate obstetric outcomes in adolescent pregnancies and contrast them with outcomes from pregnancies in older women. The study’s structured methodology adheres to the Strengthening the Reporting of Observational Studies in Epidemiology (STROBE) guidelines to ensure a thorough and reproducible research framework [14].

### 2.2. Settings

The range of data analyzed spanned from 1 January 2016 to 30 June 2024. Throughout this period, all adolescent patients who gave birth, as well as selected older patients, were incorporated into this study. The research was carried out in the Department of Gynecology, Obstetrics, Oncological Gynecology, Pediatric and Adolescent Gynecology at Bonifraters Medical Center, Katowice, Poland. The compiled data were evaluated by the personnel of the Chair and Department of Gynecology, Obstetrics, and Oncological Gynecology at the Medical University of Silesia in Katowice, a premier institution in maternal–fetal healthcare for adolescent patients.

### 2.3. Participants

The study cohort was divided into two principal groups: adolescent patients, defined as females up to 19 years old, and a control group comprising all other pregnant women. Furthermore, the study population was subdivided into three age categories: Group I (participants younger than 19 years old), Group II (participants aged 20–39 years), and Group III (participants aged 40–47 years). The exclusion criteria were chronic diseases present before pregnancy and multiple gestations. The exclusion criteria included chronic diseases present before pregnancy, such as diabetes, hypertension, congenital thrombophilia, psoriasis, hypothyroidism, ulcerative colitis, and asthma. Additionally, one of the exclusions was multiple gestations. In the group of adolescent patients, 50 patients were excluded due to chronic diseases, which accounted for 17.8% of all adolescent patients. In the group of patients aged 40–47, 86 were excluded due to chronic diseases, representing 28.3% of the patients.

### 2.4. Variables

The variables were grouped into maternal, pregnancy, birth, and newborn characteristics, and are included in Table 1.

### 2.5. Data Sources

The dataset comprises hospital medical records. The data were stored using Mediqus software (version 4.052.J.3; license WMS Mediqus Szpital; Gabos, Poland) and KS-Medis software (version 2024.02.3.0; license 2142PI01.00; Kamsoft, Poland). All patient data were anonymized and processed in accordance with relevant regulations. The data were extracted from the system by independent researchers. Additionally, two researchers (M.M-D. and J.S.) independently analyzed and extracted the anonymized data.

### 2.6. Bias

The issue of missing data can introduce bias and compromise the validity of study results. To minimize this risk, incomplete patient records were excluded from the analysis. Additionally, it is important to note that the researchers who extracted the data from the system were blinded to the ages of the patients. This measure was taken to prevent any potential bias that could arise from knowing the patients’ ages during data extraction, thereby ensuring the objectivity and integrity of this study’s findings.

### 2.7. Study Size

**Figure 1:**
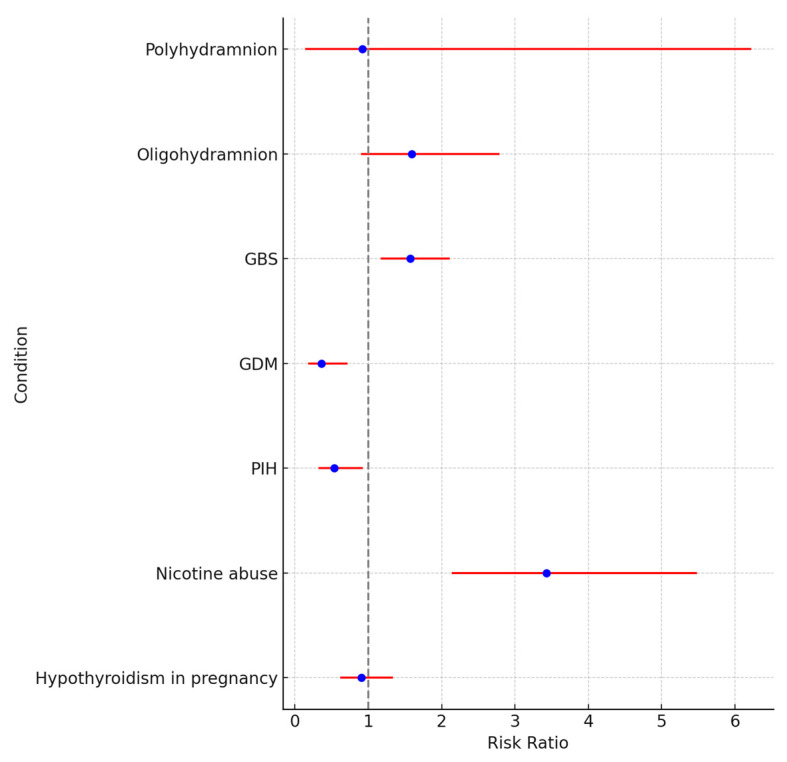
A forest plot of risk ratio for maternal and fetal outcomes.

During the study period, there were 14,066 deliveries in our hospital, of which 281 involved adolescent pregnancies (under 19 years of age) and 304 involved individuals of advanced reproductive age (40–47 years). After excluding patients who did not meet the inclusion criteria and those with incomplete documentation, this study included 224 adolescent patients and 212 patients of advanced reproductive age. A control group of 484 patients aged 20–39 years was included in this study, randomly selected from those meeting the inclusion criteria based on the ICD-10 and ICD-9 codes by the hospital’s medical statistics department. A flowchart of the inclusion and exclusion of participants is presented in Figure 1.

### 2.8. Statistics Methods

All statistical analyses were conducted using the R language in the RStudio environment. To compare qualitative variables across three groups of patients, the chi-square test was applied. Prevalence (P) was expressed as the number of cases, and confidence intervals (95% CIs) were calculated to assess the precision of estimations. Comparisons between groups were made using the proportion test, with *p*-values also calculated for pairwise comparisons (Group I vs. Group II, Group I vs. Group III). To control for multiple comparisons, we applied the Benjamani–Hochberg procedure to adjust the *p*-values and control the False Discovery Rate (FDR). Risk ratios (RRs), along with 95% confidence intervals (95% CIs) were computed to estimate the risk of complications in teenagers compared to the reference group of women aged 20–35 years, using logistic regression models. On the charts, we applied a logarithmic scale to the X-axis to improve the visibility and interpretability of the data, particularly where there were wide-ranging values. This adjustment allows for a clearer representation of both small and large risk ratios, accommodating extreme values without compressing the majority of the data points into a small section of the chart. All statistical tests were two-sided, and the level of statistical significance was set at *p* < 0.05.

## 3. Results

### Participants Characteristics

The general characteristics of the study groups are summarized in Table 2.

Group I consisted of individuals aged 14 to 19, with a sample size of 224 participants. The mean age in this group was 17.9 years, with an average height of 163.4 cm and an average weight of 67.8 kg, resulting in a mean BMI of 25.4. In this group, 92.41% were primiparous, while 7.59% were multiparous. The average gestation period at birth was 39.1 weeks, and 18 participants experienced preterm births.

Group II included 484 participants aged 20 to 39. The mean age was 30.35 years, with an average height of 165.45 cm and a mean weight of 79.55 kg, leading to a mean BMI of 29.1. In this group, 40.29% were primiparous and 59.71% were multiparous. The average gestation period was 38.45 weeks, with 27 participants having preterm births.

Group III comprised individuals aged 40 to 47, with a sample size of 212 participants. The mean age was 42.1 years, the mean height was 165.4 cm, and the average weight was 78.2 kg, resulting in a mean BMI of 27.7. In this group, 12.26% were primiparous, while 87.74% were multiparous. The mean gestation period was 38.1 weeks, and 16 participants had preterm births.

When comparing the three groups, there were significant differences in the proportions of primiparous and multiparous individuals. The *p*-value was <0.001, indicating a statistically significant difference between the groups, suggesting that a woman’s age strongly influences whether she is primiparous or multiparous.

In terms of pelvic dimensions, there were slight variations across the groups. Group I had the smallest mean measurements for distantia spinarum (25.5 cm), distantia cristarum (28.5 cm), and conjugata externa (19.1 cm). Group III showed the largest values for distantia cristarum (29.4 cm) and distantia trochanterica (32.2 cm), reflecting potential differences in pelvic morphology related to age.

## 4. Prevalence

A study comparing the prevalence of outcomes in adolescent pregnancy and control groups revealed several key differences.

Pregnancy-induced hypertension was less common among adolescents (6.70%, 95% CI: 3.42–9.97), with higher rates observed in Group II (12.60%, 95% CI: 9.65–15.56) and Group III (15.57%, 95% CI: 10.69–20.45) (*p* = 0.013, FDR = 0.037). Similarly, hypothyroidism in pregnancy showed a comparable pattern, affecting 13.84% (95% CI: 9.32–18.36) of adolescents, compared to 15.29% (95% CI: 12.08–18.50) in Group II and 24.53% (95% CI: 18.74–30.32) in Group III (*p* = 0.004, FDR = 0.020). Gestational diabetes mellitus was significantly less prevalent in adolescents (3.57%, 95% CI: 1.14–6.00) compared to Group II (10.54%, 95% CI: 7.80–13.27) and Group III (14.15%, 95% CI: 9.46–18.84) (*p* = 0.001, FDR = 0.007). Nicotine abuse was markedly more common in adolescents, with 17.86% (95% CI: 12.84–22.87) of pregnant adolescents reporting tobacco use, compared to 5.17% (95% CI: 3.19–7.14) in Group II and 4.25% (95% CI: 1.53–6.96) in Group III (*p* < 0.001, FDR = 0.001). Group B Streptococcus infection was also significantly more prevalent in adolescents (25.89%, 95% CI: 20.16–31.63), in comparison to 16.53% (95% CI: 13.22–19.84) in Group II and 17.92% (95% CI: 12.76–23.09) in Group III (*p* = 0.012, FDR = 0.037).

Labor induction was less frequently performed in adolescents (28.42%, 95% CI: 17.69–28.74) compared to Group II (33.26%, 95% CI: 29.07–37.46) and was significantly more common in Group III (72.68%, 95% CI: 56.23–69.24) (*p* < 0.001, FDR = 0.001). However, failure of labor induction was reported in 40.38% (95% CI: 5.56–13.19) of adolescents, compared to 49.07% (95% CI: 13.03–19.61) in Group II and 39.10% (95% CI: 18.74–30.32) in Group III (*p* < 0.001, FDR = 0.019). Blood transfusions were slightly more frequent among adolescents (3.13%, 95% CI: 0.85–5.40), in comparison to 0.62% (95% CI: 0.0–1.32) in Group II and 0.94% (95% CI: 0.0–2.24) in Group III (*p* = 0.021, FDR = 0.053).

Newborn complications were slightly more frequent in adolescents (20.09%, 95% CI: 14.84–25.34) compared to 14.88% (95% CI: 11.71–18.05) in Group II and 22.17% (95% CI: 16.58–27.76) in Group III (*p* = 0.041, FDR = 0.091). Admission to neonatal intensive care was significantly more common in adolescents (2.68%, 95% CI: 0.56–4.79) than in Group II (0.21%, 95% CI: 0.0–0.61) and Group III (0.94%, 95% CI: 0.0–2.24) (*p* = 0.008, FDR = 0.028).

The prevalence of obstetric and neonatal parameters across three distinct age groups is presented in Table 3.

## 5. Risk Ratios (RRs) for Obstetric and Neonatal Outcomes in Adolescent Pregnancy

The risk ratios (RRs), 95% confidence intervals (CIs) for RR, *p*-value, and False Discovery Rate (FDR) for obstetric and neonatal outcomes in adolescent pregnancy are presented in Table 4.

### 5.1. Maternal Outcomes

Oligohydramnios presents a high risk, with an RR of 1.59 (95% CI: 0.90–2.79). The presence of Group B Streptococcus (GBS) infection significantly increases the risk, with an RR of 1.57 (95% CI: 1.17–2.11). Gestational diabetes mellitus (GDM) has a reduced risk, indicated by an RR of 0.36 (95% CI: 0.18–0.72), and Pregnancy-Induced Hypertension (PIH) also shows a lower risk, with an RR of 0.54 (95% CI: 0.32–0.93). Nicotine abuse is associated with a notably higher risk, with an RR of 3.43 (95% CI: 2.14–5.48). A forest plot for maternal and fetal outcomes is presented in Figure 1.

### 5.2. Birth Outcomes

**Figure 2:**
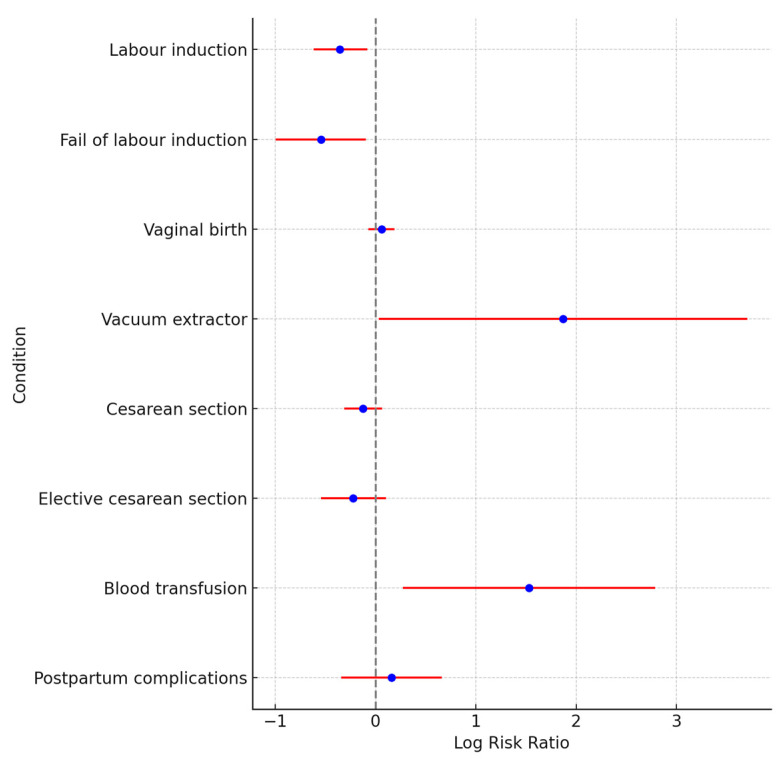
A forest plot of risk ratio for birth outcomes.

Labor induction is associated with a lower risk, with an RR of 0.70 (95% CI: 0.54–0.92), and the risk of failure of labor induction is also lower, with an RR of 0.58 (95% CI: 0.37–0.91). The use of a vacuum extractor shows a substantially higher risk, with an RR of 6.47 (95% CI: 1.03–40.81). The risk for cesarean section is slightly lower, with an RR of 0.88 (95% CI: 0.73–1.07), and elective cesarean section shows a decreased risk, with an RR of 0.80 (95% CI: 0.58–1.11). The need for a blood transfusion significantly increases the risk, with an RR of 4.62 (95% CI: 1.31–16.29). A forest plot for birth outcomes is presented in Figure 2.

### 5.3. Newborn Outcomes

**Figure 3:**
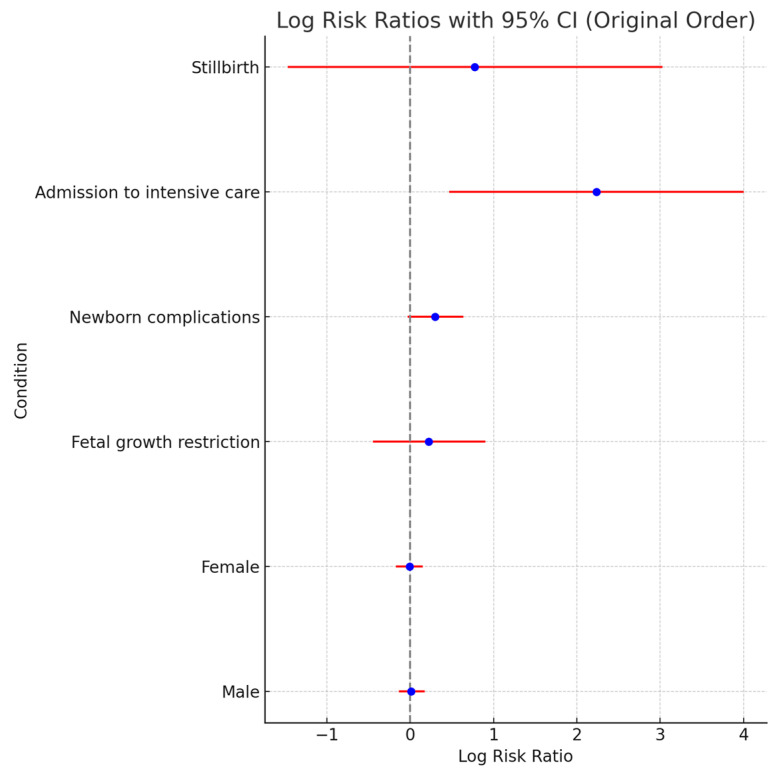
A forest plot of risk ratio for newborn outcomes.

Fetal growth restriction shows a moderately higher risk, with an RR of 1.25 (95% CI: 0.64–2.47), and the risk of newborn complications is slightly increased, with an RR of 1.35 (95% CI: 0.97–1.89). Admission to intensive care shows a substantial increase in risk, with an RR of 9.35 (95% CI: 1.60–54.81). A forest plot for newborn outcomes is presented in Figure 3.

## 6. Discussion

This study highlights the increased risks associated with adolescent pregnancies, particularly regarding maternal and neonatal complications. Our findings align with the existing literature.

We observed a significantly higher prevalence of Group B Streptococcus (GBS) infection among adolescent mothers (25.89%; 95% CI: 20.16–31.63%), which was notably higher than in older age groups (*p* = 0.012). This is consistent with the findings of Maheshwari et al. [5], who also identified an elevated susceptibility to infections, including GBS, among adolescent mothers. The increased risk of GBS infection is concerning, as it can lead to severe complications such as preterm labor and neonatal sepsis [15,16].

Nicotine abuse was significantly higher in adolescent pregnancies, with 17.86% (95% CI: 12.84–22.87%) of adolescents reporting smoking during pregnancy (*p* < 0.001). This finding echoes the results of studies such as those by Salas-Wright et al. [17], which highlighted the higher prevalence of smoking among pregnant adolescents. The adverse effects of nicotine, including fetal growth restriction and low birth weight, are well-documented, emphasizing the importance of targeted smoking cessation programs for this age group. Aliyu et al. [18] also show that the risk of intrapartum stillbirth among smoking adolescents under 15 years of age was twice the risk for older adolescent and mature mothers. The risk of intrapartum stillbirth among smokers decreased as maternal age increased: [adjusted hazard ratio (AHR), 95% confidence interval (CI) for young mothers: 4.0, 95% CI = 0.6–28.7; for older adolescents AHR = 1.5, 95% CI = 1.1–2.1; and for mature mothers AHR = 1.8, 95% CI = 1.4–2.2].

Additionally, this study highlighted the significant blood loss associated with deliveries among adolescent mothers. The mean blood loss during vaginal births was 500 mL, and during cesarean sections, it was 1050 mL, both figures notably higher than those in older age groups. This contributed to a higher incidence of blood transfusions in adolescent mothers (3.13%; 95% CI: 0.85–5.40%; *p* = 0.021). This finding is supported by the work of Brosens et al. [19], who discussed the challenges posed by the uterine immaturity of adolescent mothers, leading to increased obstetric complications, including postpartum hemorrhage.

The neonatal outcomes observed in our study further emphasize the prevalence associated with adolescent pregnancies. Neonatal complications were significantly more frequent in the adolescent group, affecting 20.09% (95% CI: 14.84–25.34%) of newborns, with a significant difference compared to the middle-aged group (*p* = 0.041). This is in line with the findings of Azevedo et al. [10], who also reported higher rates of neonatal complications among children born to adolescent mothers. The increased need for neonatal intensive care unit (NICU) admission (2.68%; 95% CI: 0.56–4.79%; *p* = 0.008) further underscores the vulnerability of this population.

Interestingly, despite the lower rate of labor induction in adolescent pregnancies (28.42%; 95% CI: 17.69–28.74%), the cesarean section rates were similar across age groups (*p* = 0.355). This observation is consistent with studies such as those by Malabarey et al. [20], which also found no significant age-related differences in cesarean delivery rates. The statistically significant differences in labor induction rates (*p* < 0.001) suggest that younger patients were less likely to undergo medical interventions such as labor induction. However, when induction was performed, they had the lowest failure rate (40.38%), with statistically significant differences (*p* < 0.002 for Group I vs. Group II and *p* < 0.001 for Group I vs. Group III). Our data suggest that younger pregnant women are less frequently subjected to labor induction but do not experience significantly worse outcomes in terms of postpartum complications when induction is performed. In fact, the similar rates of complications between induced and non-induced groups may indicate that young women tolerate induction well and may even benefit from it. This could potentially reduce neonatal complications if induction were utilized more frequently and proactively. This observation aligns with studies indicating that, when appropriately managed, induction does not increase the rate of cesarean sections and may help prevent certain complications [21].

The findings also indicate that bilirubin levels in the newborns of adolescent patients 24 h postpartum were significantly higher (7.66 mg/dL) compared to the control groups. Notably, primiparous women constituted a significantly larger proportion of the adolescent group (92.41%), with this distribution difference being statistically significant (*p* < 0.001). The elevated bilirubin levels may be associated with the higher prevalence of primiparity and challenges in establishing breastfeeding among this population, consistent with the literature by Volpe et al. [22] and Leclair et al. [23]. These studies emphasize the importance of providing targeted support and education to adolescent mothers to promote successful breastfeeding initiation.

The findings of this study, while significant, should be interpreted within the context of specific limitations. Our data were derived from adolescent pregnancies in a particular geographic region and health system, which may limit the generalizability of the results to other populations. Variations in healthcare access, socioeconomic status, and health education can influence maternal and neonatal outcomes, and these factors were not fully accounted for in our study. Furthermore, the higher prevalence of certain complications, such as GBS infection and nicotine abuse, may be region-specific. The cohort size may also have impacted the statistical power, making it more challenging to detect subtle differences between groups. Future research with larger sample sizes and more extensive data collection would help validate these findings and provide a more comprehensive understanding of the risks associated with adolescent pregnancies. Despite these limitations, the consistency of our findings with other studies suggests that the increased risks associated with adolescent pregnancies are a widespread concern, highlighting the need for targeted healthcare interventions for this vulnerable group.

## 7. Conclusions

Our study confirms that adolescent pregnancy is associated with significantly increased risks for both the mother and the newborn, as evidenced by a higher incidence of complications, such as Group B Streptococcus (GBS) infections, nicotine use, excessive blood loss during delivery, and an increased number of neonatal complications. These findings are consistent with previous research, underscoring the need for more targeted interventional strategies in this age group [24]. It is essential to support and educate adolescent mothers to promote successful breastfeeding initiation, particularly since they are at a higher risk of either not starting or quickly discontinuing lactation. Our data also suggest that the use of labor induction in this population may help reduce the number of complications for both the mother and the newborn. Although labor induction is currently less common among adolescents, the results indicate that it could be considered more frequently. Therefore, a more proactive approach to labor induction in this age group should be considered while maintaining caution and tailoring the approach to the individual needs of the patient to minimize risks and improve health outcomes.

## Figures and Tables

**Table 1:** Maternal, pregnancy, birth, and newborn variables.

Category	Variable
Maternal Variables	Ethnicity
	Parity
	Group B Streptococcus (GBS)
	Gestational diabetes mellitus (GDM)
	Pregnancy-Induced Hypertension (PIH)
	Hypothyroidism in pregnancy
	Nicotine abuse
	Pelvic dimensions (cm)
	Postpartum complications were defined as the occurrence of one of the following in the mother: perineal trauma, the need for episiotomy, postpartum hemorrhage, infection (such as endometritis or wound infection), uterine inversion, shoulder dystocia, uterine atony, retained placenta requiring manual removal, severe anemia requiring blood transfusion, deep vein thrombosis, and obstetric anal sphincter injuries (OASIS).
Pregnancy Variables	Polyhydramnion
	Oligohyramnion
	Fetal growth restriction
Birth Variables	Preterm birth (<37 weeks of pregnancy)
	Labor induction
	Fail of labor induction
	Hemoglobin before birth (mean, mg/dL)
	Hemoglobin after birth (mean, mg/dL)
	Blood loss birth (mean, mL)
	Blood loss cesarean section (mean, mL)
	Blood transfusion
	Postpartum complications
	Vaginal birth
	Vacuum extractor
	Cesarean section
	Elective cesarean section
Newborn Variables	Newborn sex
	Newborn weight (g)
	Newborn length (cm)
	APGAR score (mean, points)
	Bilirubin after 24 h (mean, mg/dL)
	Newborn complications were defined as the occurrence of one of the following in the newborn: an APGAR score < 7, pH < 7.0, BE −16 mmol/L, at least Stage I in the Sarnat Score, brachial plexus injury (e.g., Erb’s palsy), skull fractures, caput succedaneum, subconjunctival hemorrhage, intracranial hemorrhage (subgaleal hematoma, epidural hematoma, or cephalohematoma), parenchymal organ hemorrhage (liver, spleen, kidney, or adrenal hematoma), facial nerve injury, soft tissue injuries (such as severe bruising or lacerations), fractures of long bones, clavicle fracture, umbilical cord rupture, meconium aspiration syndrome (MAS), or early-onset neonatal sepsis (EOS).
	Discharge to home (mean, day)
	Admission to intensive care
	Stillbirth

**Table 2:** The general characteristics of the study groups.

	GROUP I: 14–19	GROUP II: 20–39	GROUP III: 40–47
	N = 224	N = 484	N = 212
	n/M	n/M	n/M
Age (Mean)	17.9	30.35	42.1
Ethnicity			
White	224	484	212
Height (cm)	163.4	165.45	165.4
Weight (kg)	67.8	79.55	78.2
BMI (kg/m^2^)	25.4	29.1	27.7
Primipara	207	195	26
Multipara	17	289	186
Birth gestation (weeks)	39.1	38.45	38.1
Birth gestation < 37 weeks	18	27	16
Pelvic dimentions (cm)			
Distantia spinarum	25.5	26.1	25.9
Distantia cristarum	28.5	29.15	29.4
Distantia trochanterica	31.7	32	32.2
Coniugata externa	19.1	20.65	20.9
Hemoglobin before birth (mean, mg/dL)	10.9	12.3	12.0
Hemoglobin after birth (mean, mg/dL)	9.1	11.95	11.7
Blood loss birth (mean, mL)	500	300	350
Blood loss cesarean section (mean, mL)	1050	600	700
Newborn weight (g)	3199	3325.5	3397
Newborn lenght (cm)	53.6	54.45	55
APGAR score < 7	14	25	10
Bilirubin after 24 h (mean, mg/dL)	7.66	5.875	6.01
Discharge to home (mean, day)	2.9	2.4	2.3

**Table 3:** The prevalence of obstetric and neonatal parameters across three distinct age groups.

	GROUP I: 14–19				GROUP II: 20–39				GROUP III: 40–47				*p*_Value	*p*_Value I vs. II	*p*_Value I vs. III	FDR	*p*_Value_I_vs._II	FDR I_vs._II	*p*_Value_I_vs._III	FDR I_vs._III
	N = 224				N = 484				N = 212											
	N	*p*		95% CI	N	*p*		95% CI	N	*p*		95% CI								
Polyhydramnion	1	0.45%	0	0.0132	3	0.62%	0	0.0132	2	0.94%	0	0.0224	0.806	1	0.962	0.866	1.000	1.000	0.962	1.000
Oligohydramnion	19	8.48%	0.0483	0.1213	26	5.37%	0.0336	0.0738	13	6.13%	0.029	0.0936	0.283	0.158	0.449	0.477	0.158	0.302	0.449	0.857
Fetal growth restriction	12	5.36%	0.0241	0.0831	21	4.34%	0.0252	0.0615	12	5.66%	0.0255	0.0877	0.708	0.685	1	0.866	0.685	0.860	1.000	1.000
Group B Streptococcus	58	25.89%	0.2016	0.3163	80	16.53%	0.1322	0.1984	38	17.92%	0.1276	0.2309	0.012	0.005	0.059	0.037	0.005	0.035	0.059	0.177
Gestational diabetes mellitus	8	3.57%	0.0114	0.0600	51	10.54%	0.0780	0.1327	30	14.15%	0.0946	0.1884	0.001	0.003	<0.001	0.007	0.003	0.032	<0.001	0.001
Pregnancy-Induced Hypertension	15	6.70%	0.0342	0.0997	61	12.60%	0.0965	0.1556	33	15.57%	0.1069	0.2045	0.013	0.026	0.005	0.037	0.026	0.068	0.005	0.021
Hypothyroidism in pregnancy	31	13.84%	0.0932	0.1836	74	15.29%	0.1208	0.1850	52	24.53%	0.1874	0.3032	0.004	0.696	0.007	0.020	0.696	0.860	0.007	0.025
Nicotine abuse	40	17.86%	0.1284	0.2287	25	5.17%	0.0319	0.0714	9	4.25%	0.0153	0.0696	<0.001	<0.001	<0.001	0.001	<0.001	0.002	<0.001	0.001
Labor induction	52	28.42%	0.1769	0.2874	161	33.26%	0.2907	0.3746	133	72.68%	0.5623	0.6924	<0.001	0.009	<0.001	0.001	0.009	0.038	<0.001	0.001
Fail of labor induction	21	40.38%	0.0556	0.1319	79	49.07%	0.1303	0.1961	52	39.10%	0.1874	0.3032	<0.001	0.019	<0.001	0.019	0.019	0.066	<0.001	0.001
Vaginal birth	134	59.82%	0.5340	0.6624	273	56.40%	0.5199	0.6082	128	60.38%	0.5379	0.6696	0.523	0.439	0.984	0.697	0.439	0.659	0.984	1.000
Vacuum extractor	4	1.79%	0.0005	0.0352	1	0.21%	0	0.0061	2	0.94%	0	0.0224	0.075	0.064	0.731	0.150	0.064	0.149	0.731	1.000
Cesarean section	86	38.39%	0.3202	0.4476	211	43.60%	0.3918	0.4801	84	39.62%	0.3304	0.4621	0.355	0.222	0.869	0.507	0.222	0.359	0.869	1.000
Elective cesarean section	41	47.67%	0.1324	0.2337	111	52.61%	0.1919	0.2668	50	59.52%	0.1787	0.293	0.31	0.195	0.216	0.477	0.195	0.341	0.216	0.504
Blood transfusion	7	3.13%	0.0085	0.0540	3	0.62%	0	0.0132	2	0.94%	0	0.0224	0.021	0.022	0.206	0.053	0.022	0.066	0.206	0.504
Postpartum maternal complications	21	9.38%	0.0556	0.1319	39	8.06%	0.0563	0.1048	25	11.79%	0.0745	0.1613	0.293	0.66	0.506	0.477	0.660	0.860	0.506	0.886
Newborn sex																				
Male	113	50.45%	0.4390	0.5699	241	49.79%	0.4534	0.5425	111	52.36%	0.4564	0.5908	0.823	0.936	0.762	0.866	0.936	1.000	0.762	1.000
Female	111	49.55%	0.4301	0.5610	243	50.21%	0.4575	0.5466	101	47.64%	0.4092	0.5436	0.823	0.936	0.762	0.866	0.936	1.000	0.762	1.000
Postpartum newborn complications	45	20.09%	0.1484	0.2534	72	14.88%	0.1171	0.1805	47	22.17%	0.1658	0.2776	0.041	0.104	0.678	0.091	0.104	0.218	0.678	1.000
Admission to intensive care	6	2.68%	0.0056	0.0479	1	0.21%	0	0.0061	2	0.94%	0	0.0224	0.008	0.007	0.321	0.028	0.007	0.037	0.321	0.674
Stillbirth	1	0.45%	0	0.0132	1	0.21%	0	0.0061	2	0.94%	0	0.0224	0.397	1	0.962	0.962	1.000	1.000	0.962	1.000

**Table 4:** Risk ratios (RRs), 95% confidence intervals (CIs) for RR, *p*-value, and False Discovery Rate (FDR) for obstetric and neonatal outcomes in adolescent pregnancy.

	RR	95% CI RR		*p*-Value	FDR
Polyhydramnion	0.92	0.14	6.22	0.934	0.936
Oligohydramnion	1.59	0.90	2.79	0.107	0.224
Fetal growth restriction	1.25	0.64	2.47	0.512	0.715
Group B Streptococcus	1.57	1.17	2.11	0.004	0.033
Gestational diabetes mellitus	0.36	0.18	0.72	0.004	0.033
Pregnancy-Induced Hypertension	0.54	0.32	0.93	0.025	0.073
Nicotine abuse	3.43	2.14	5.48	<0.001	<0.001
Hypothyroidism in pregnancy	0.91	0.62	1.34	0.642	0.805
Labor induction	0.70	0.54	0.92	0.01	0.055
Fail of labor induction	0.58	0.37	0.91	0.019	0.062
Vaginal birth	1.06	0.93	1.21	0.379	0.622
Vacuum extractor	6.47	1.03	40.81	0.048	0.119
Cesarean section	0.88	0.73	1.07	0.208	0.366
Elective cesarean section	0.80	0.58	1.11	0.178	0.343
Blood transfusion	4.62	1.31	16.29	0.017	0.062
Postpartum complications	1.17	0.71	1.94	0.529	0.715
Male	1.01	0.87	1.19	0.177	0.924
Female	0.99	0.84	1.16	0.885	0.924
Newborn complications	1.35	0.97	1.89	0.077	0.175
Admission to intensive care	9.35	1.60	54.81	0.013	0.061
Stillbirth	2.16	0.23	20.63	0.504	0.715

## Data Availability

The original contributions presented in the study are included in the article, further inquiries can be directed to the corresponding author.
